# Negative and Positive Bias for Emotional Faces: Evidence from the Attention and Working Memory Paradigms

**DOI:** 10.1155/2021/8851066

**Published:** 2021-05-27

**Authors:** Qianru Xu, Chaoxiong Ye, Simeng Gu, Zhonghua Hu, Yi Lei, Xueyan Li, Lihui Huang, Qiang Liu

**Affiliations:** ^1^Institute of Brain and Psychological Sciences, Sichuan Normal University, Chengdu, China; ^2^Department of Psychology, University of Jyvaskyla, Jyväskylä, Finland; ^3^Department of Medical Psychology, Jiangsu University Medical School, Zhenjiang, China; ^4^School of Foreign Languages, Dalian University of Technology, Dalian, China; ^5^Faculty of Education, Sichuan Normal University, Chengdu, China

## Abstract

Visual attention and visual working memory (VWM) are two major cognitive functions in humans, and they have much in common. A growing body of research has investigated the effect of emotional information on visual attention and VWM. Interestingly, contradictory findings have supported both a negative bias and a positive bias toward emotional faces (e.g., angry faces or happy faces) in the attention and VWM fields. We found that the classical paradigms—that is, the visual search paradigm in attention and the change detection paradigm in VWM—are considerably similar. The settings of these paradigms could therefore be responsible for the contradictory results. In this paper, we compare previous controversial results from behavioral and neuroscience studies using these two paradigms. We suggest three possible contributing factors that have significant impacts on the contradictory conclusions regarding different emotional bias effects; these factors are stimulus choice, experimental setting, and cognitive process. We also propose new research directions and guidelines for future studies.

## 1. Introduction

In the processing of visual information, attention and memory are two cognitive processes that play pivotal roles in human life, and they are extremely important aspects of psychology and cognitive neuroscience research. Previously, however, these two topics have been studied separately; for example, memory studies have not tended to explore the effect of selective attention on memory encoding, while attention studies have often neglected the consequence of past experience [1]. In recent years, a growing body of research has begun to explicitly link visual attention to visual working memory (VWM, which could also be called “visual short-term memory,” VSTM). These studies have reached a broad consensus that attention and VWM are intimately linked [2–4]. This consensus is unsurprising, given that the definitions of “attention” and “VWM” already overlap significantly.

As defined by Olivers et al. [2], visual attention describes a process during which individuals select relevant information and ignore irrelevant information. By contrast, VWM describes the process during which individuals temporarily retain relevant information and suppress irrelevant information. In addition to the similarity of their definitions, the visual attention and VWM processes may have many overlapping mechanisms, such as the activation of many similar brain regions (e.g., the supplementary motor area and frontal eye fields, the lateral prefrontal cortex, the anterior cingulate, the superior and inferior parietal cortex, and the occipital area) and a similar capacity limitation (for about four units or chunks), as well as similar control processes (for a review, see [3]). Therefore, exploring the relationship between visual attention and VWM is highly significant for obtaining a better understanding of basic human cognition [5–11].

Emotional processing, another major cognitive function for humans, has attracted considerable interest in both the visual attention and VWM fields. Regarding visual attention, many studies have examined attentional bias toward emotional stimuli, which can be further divided into negative bias and positive bias (for negative bias, see [12–15]; for positive bias, see [16–18]; for reviews, see [19, 20]). (The phenomenon of negative and positive bias has been studied extensively using a variety of emotional materials, such as faces, scenes, and words [19, 21]. However, we mainly focus in this paper on previous studies that have used emotional faces for the following reasons. First, humans are experts in assessing faces [22]. Compared to other stimuli, faces more easily attract visual attention, and they are more likely to be stored in the human VWM than other complex stimuli [23]. Second, the same facial identity can reflect different types of emotions with little physical difference between the emotions, while other emotional stimulus materials (e.g., different emotional scenes) differ greatly in physical features between emotions [24]. Finally, due to the short history of researching VWM as such [25, 26], the study of the emotional bias effect on VWM began only decades ago, mostly using emotional faces as materials [27–29].) “Negative bias” refers to the processing advantage of negative stimuli (e.g., angry, fearful, sad, or disgusted faces) over positive stimuli (i.e., happy faces); conversely, a “positive bias” refers to the preference for positive stimuli (i.e., happy faces) in emotional processing [19, 21]. Interestingly, VWM studies have revealed a similar phenomenon, finding both negative and positive advantages to VWM performance (for negative bias, see [27, 28, 30, 31]; for positive bias, see [32–34]). These controversial results are derived mainly from two kinds of paradigms, namely, the *visual search paradigm* in visual attention studies and the *change detection paradigm* in VWM studies. Some previous review papers have discussed the contradictory findings of previous visual attention studies (e.g., [19, 20, 35–37]). However, to our knowledge, no studies have yet combined the findings of visual attention studies with those of VWM studies to discuss the possible factors that have contributed to their contradictory outcomes. Therefore, in this paper, we conduct a literature review on previous studies that have investigated the different emotional bias effects in (a) visual attention studies using the visual search paradigm and (b) VWM studies using the change detection paradigm. Our purposes in conducting this work are to list the distinct behavioral and neural levels of evidence, to discuss the possible reasons behind the existing controversial results, and to provide new guidelines and suggestions for future emotional bias studies.

## 2. Controversial Results in Different Expressions

### 2.1. Behavior and Neural Evidence with Different Emotional Faces in the Visual Search Paradigm

#### 2.1.1. Negative Bias

**Figure 1:**
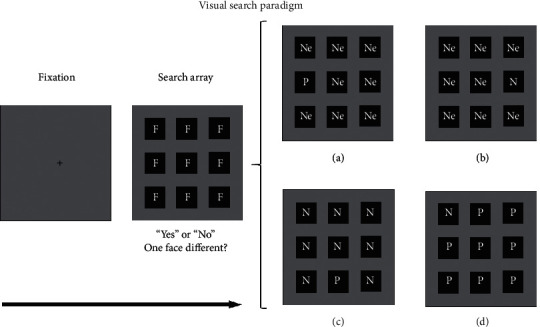
Illustration of a visual search paradigm. Participants needed to detect whether one face differed from the other faces. The letter F denotes a face in the search array. Usually, in half of the trials, all faces show the same expression, while in the other half of the trials, one face shows a different expression from the other faces. The trials containing different kinds of expressions (as presented in panels (a)–(d)) have usually occurred in four versions: (a) one positive face with a neutral face background (P: positive face; Ne: neutral face); (b) one negative face with a neutral face background (N: negative face; Ne: neutral face); (c) one positive face with a negative background (P: positive face; N: negative face); (d) one negative face with a positive background (N: negative face; P: positive face). Note that the set size in each search array can differ across studies. Negative face: angry, fearful, sad, or disgusted expression face; positive face: happy expression face; neutral face: neutral expression face.

With their use of a visual search paradigm, Hansen and Hansen [12] first found an attentional bias toward angry faces presented as black-and-white photographs, with the bias reflected in a shorter response time (RT) and a lower error rate for angry faces versus happy and neutral faces (see Figure 1 for an illustration of the stimulus conditions; see Supplementary Materials for more detailed introduction of this paradigm and frequently used behavioral and neural indexes). However, this result soon met with challenges from other studies because of the extraneous dark areas in Hansen and Hansen's black-and-white stimuli [38]. Nevertheless, even with better control of the stimuli, some follow-up studies still found an attentional bias toward angry faces (e.g., [15, 39, 40]). In addition to angry faces, fearful faces (commonly referred to as “threatening faces”—together with angry faces) have been suggested to have a similar automatic attention capture as angry faces [39]. Indeed, a fearful face seems even easier to detect than an angry face [41]. The attentional bias toward angry and fearful faces, taken together, has been called the “threat superiority effect.” This threatening bias is more widely validated by schematic face studies (e.g., [13, 42, 43]) than by studies using photographs of real faces. However, some studies have suggested that the attentional bias toward threatening faces in schematic experiments was actually an attentional bias to sad faces because the participants were more likely to label the corresponding stimulus material as “sad faces” [13].

In addition to behavioral studies, studies using other techniques have also supported the threat superiority effect. Using the eye tracking technique—which allows for relatively direct and continuous measurement of overt visual attention—a previous study using schematic faces found that, in the context of neutral faces, participants took a longer time and more fixations to fixate on the emotional face target if it was a positive face versus a negative face [44]. Another study using photographs found that participants fixated on more distractors before first fixating on a happy face target compared to an angry face target [45]. The use of electroencephalogram (EEG) technology in previous studies confirmed that angry face targets induced earlier and greater N2pc (N2-posterior-contralateral) than did happy face targets [46]. An enhanced contralateral delay activity (CDA) (also known as sustained posterior contralateral negativity [SPCN]) then indicated that angry faces might involve more subsequent processing than was required for happy faces. Moreover, lateralized early posterior negativity (EPN) showed that angry faces already induced greater negativity than happy faces at 160 ms, indicating early threat-relevant information processing.

#### 2.1.2. Positive Bias

Although early research found evidence supporting the bias toward happy faces, this phenomenon has not received sufficient attention. Most studies tended to regard it as a perceptual confounder rather than an emotional factor (see, e.g., [16]). However, further accumulation of relevant evidence [17, 18, 47–49] has renewed interest in this phenomenon. For example, Becker et al. [18] used photographs and realistic computer-graphic faces to control all the confounding variables that have arisen in previous attentional bias studies, and they found no support for efficiently detecting angry faces; however, they did find a robust positive bias effect across seven experiments. They suggested that the positive bias in their studies could not be attributed to low-level visual confounders [18]. Unlike the negative bias, which yielded a robust effect with schematic stimuli, little evidence supported the positive bias with schematic faces [19]. Only one study showed a positive bias when the distractors were changed to a heterogeneous (i.e., using different identities in the search array) background instead of a homogenous (i.e., using the same identity in the search array) background [50].

Similarly, several other neuroscience studies have supported the positive bias. For example, studies using the eye-tracking technique have provided evidence for an attentional bias toward happy faces. Calvo et al. [48], in their study, showed that happy targets were detected faster than any other expressions (e.g., surprised, disgusted, fearful, angry, or sad). Conversely, and in contrast to previous studies [44, 45], angry faces were detected more slowly and less accurately than were happy, surprised, disgusted, and fearful faces [48]. However, compared to studies on the search advantage of angry faces, fewer EEG studies have supported a bias toward happy faces, which only indirect evidence has implied. For example, one study [51] suggested that the widely used stimuli in previous studies (e.g., happy and angry faces) are not equal in biological relevance to observers. Therefore, the authors used baby faces as positive stimuli and compared the results with angry adult faces (as negative stimuli) in an attention task. Their results indicated that positive and negative stimuli induced similar modulations in P1 amplitude and with corresponding topography and source localization, suggesting that both positive and negative stimuli have similar advantages in capturing attention at the neural level [51].

### 2.2. Behavior and Neuroscience Evidence with Different Emotional Faces in the Change Detection Paradigm

#### 2.2.1. Negative Bias

**Figure 2:**
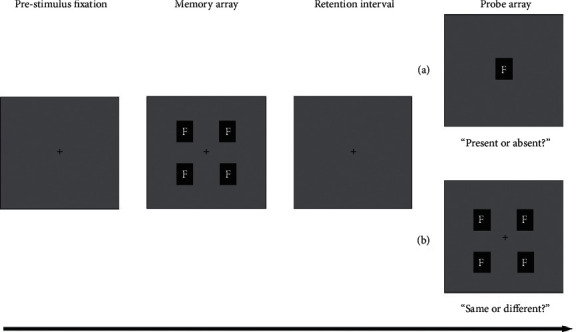
Two versions of the change detection paradigm. Participants need to detect (a) whether the single probe is present or absent in the memory array or (b) whether the probe array is identical to the memory array or one of the faces has changed. The letter F denotes a face, which can be emotional (positive or negative) or neutral in different studies. Note that the set size in the search array can differ across studies. Negative face: angry, fearful, sad, or disgusted expression face; positive face: happy expression face; neutral face: neutral expression face.

Using the change detection paradigm (see Figure 2 for an illustration of the stimulus conditions; see Supplementary Materials for more detailed introduction of this paradigm and frequently used behavioral and neural indexes), Jackson et al. [27] first examined how expression and identity interact with one another (face identity was task relevant while expression was task irrelevant). Their results consistently showed enhanced VWM performance with different set sizes, durations, and face sets. With schematic faces, other researchers limited the cognitive resources by manipulating the encoding time and set size, and they found better memory performance for angry faces with short exposure time (150 ms) and a large set size of stimuli (five items) [52]. Similarly, researchers found that participants could better maintain fearful faces in VWM than they could retain neutral faces [30, 53]. Research has also shown enhanced VWM storage for fearful faces compared to neutral faces [30, 54].

The use of EEG confirmed that threatening faces (both fearful and angry faces) showed an enhanced N170 response and higher theta power compared to both positive faces (very happy and somewhat happy faces) and neutral faces, both at the encoding stage and at the early maintenance interval after the memory array disappeared [55]. Sessa et al. [30] found that fearful faces showed an enhanced CDA compared to neutral faces, which suggested an increased maintenance for a fearful face in VWM than for a neutral face. With a similar experimental setting as their own study, Jackson et al. [28] found the results of functional magnetic resonance imaging (fMRI) supported a benefit of angry faces in the change detection paradigm. Compared to happy and neutral faces, angry faces significantly enhanced blood oxygen level-dependent responses—particularly in three areas of the right hemisphere: the prefrontal cortex, the superior temporal sulcus, and the globus pallidus internus [28].

#### 2.2.2. Positive Bias

Although initial studies have generally reported a negative bias in VWM, the happy face benefit (or threatening face cost) has appeared in recent studies [32–34, 53, 56]. One study that used photographs [53] found superior memory sensitivity for fearful faces but also for happy faces compared to neutral faces. Interestingly, by manipulating memory array and encoding time, Curby et al. [34] found worse VWM performance for fearful faces than for neutral and happy faces, which suggested a fearful face cost in VWM compared to happy and neutral faces. The addition of location information to the change detection paradigm also revealed that the relocation accuracy for happy faces was significantly enhanced compared to angry faces [33]. Studies using schematic faces have also found that, although no memory differences occurred between different emotional faces (approach-oriented positive faces versus avoid-oriented negative faces), high-capacity participants tended to maintain more positive (e.g., happy) than negative (e.g., sad/angry) faces, and this was reflected in a significant correlation between affective bias and the participants' VWM capacity [32].

However, as with the attention studies, the positive advantage in VWM has found less support from neuroscientific evidence. Compared to happy faces, sad faces tend to significantly attenuate facial identity recognition, a finding supported by the exhibited components of N170, N250, P3b, vertex positive potential, and late positive potential [57]. This finding can be partially verified by the overall emotional advantage effect. For example, using the EEG technique, researchers examined the event-related potential (ERP) components of P1, N170, P3b, and N250r in a VWM task [58]. Their results showed that none of these ERP components were modulated by emotional faces during the encoding stage. During maintenance, a decreased early P3b and increased N250r for emotional faces were observed when compared to neutral faces, but no difference in ERP components was apparent between positive and negative faces.

Overall, the development processes and evidence patterns of the change detection paradigm and visual search paradigm are quite similar. The findings of a negative bias have a relatively longer history and greater support from empirical research using cognitive neuroscience techniques. By contrast, the findings of a positive bias have mostly resulted from recent behavioral studies with better control over the potential confounding variables. However, scant neuroscience evidence has supported the positive bias for either the attentional or the VWM studies.

## 3. Possible Contributing Factors for Emotional Bias

The findings above show that both attention and VWM studies have revealed some controversial results regarding emotional bias. Some studies have discussed and listed several potential contributors for the emotional bias in attention (e.g., [19, 20, 35, 36]). However, to the best of our knowledge, no study has summarized the positive and negative face advantages in VWM. Therefore, we have summarized and listed these advantages in Supplementary Table 1 (including 20 papers with 36 experiments), especially regarding the adoption of the change detection paradigm [27, 28, 30–34, 52, 53, 55–65]. Based on the table summarized by previous studies on visual attention (see [18] for a summary of the visual search paradigm; see [19] for more general methods) and our table for VWM (see Supplementary Material Table 1), we found some common factors responsible for the contradictions in these two areas—especially for studies using the visual search and change detection paradigms. Below, we discuss these possible contributing factors separately, using three aspects: stimulus choice, experimental setting, and cognitive process.

### 3.1. Differences in Stimulus Choice

In both visual search and change detection paradigm, the experimental materials used for different studies often differ. Previous controversial results could therefore simply reflect the different choices in stimulus materials.

#### 3.1.1. Schematic Faces versus Real Faces

Both photographs of real faces and schematic faces are widely used stimuli in the visual search and change detection paradigms. However, a more consistent negative bias occurs with schematic faces, while photographs of real faces show more evidence of a positive bias for visual attention (for reviews, see [19, 37]). Thus, the choice of stimulus (schematic or real faces) used in an experiment is crucial. Similarly, in the field of VWM, as we mentioned in the previous section, different studies using different stimuli have yielded different results.

For visual attention, a schematic face undoubtedly allows for better control of physical features than can be achieved with photographs. However, the representative expressions of a schematic face are limited, and they lack ecological validity. Thus, schematic faces have been criticized for presenting differences in the perceived configuration of the stimulus itself, rather than reflecting a direct response to emotions [66–68]. For example, some researchers have emphasized that the attentional bias toward angry faces in the visual search paradigm using schematic faces resulted from perceptual grouping, in which participants perceived happy faces as a group more easily than angry faces; therefore, angry faces were more salient when happy faces served as distractors [68]. Photographs of real faces are more ecologically valid; however, the results differ significantly for visual search studies. Previous studies have even found different results based on individual differences and different stimulus sets as the materials in the visual search paradigm [69]. Moreover, when using photographs, various settings of the eyes and mouth may be potential influencing factors. For example, emotional bias can be obtained from the eye characteristics alone (for bias toward angry faces, see [70]; for bias toward happy faces, see [18]). Whether the teeth are exposed also leads to different results as well [71]. However, these factors undeniably also serve as the major composition of the expression per se; thus, one cannot entirely attribute this controversy to perceptual differences, especially for photographs.

Similarly, in the change detection paradigm, the results for schematic faces have also tended to favor either a negative bias or an overall affective bias, which may also relate to problems that we mentioned earlier in attention studies. Different studies using photographs have used various sets of stimulus materials (see Supplementary Table 1). For example, the series of experiments by Jackson et al. [27] used the Ekman set [72] and the Karolinska Directed Emotional Faces (KDEF) database [73], while the materials used by Curby et al. [34] were a collection of four stimulus databases (the NimStim database [74], the KDEF database [73], the CVL Face Database [75], and the Radboud Faces Database [76]). These variations in stimulus materials from different studies complicate any direct comparison of the two effects. Besides, the stimuli used in previous studies did not rule out the effect of some subtle issues that we mentioned above, such as potential influences from the eyes or mouth regions. Although we cannot conclude that different results are due to the use of different stimuli (e.g., the study by Jackson et al. [27] validated an angry face advantage in both image databases), neither can we completely reject the possibility that different memory advantages are irrelevant to the choice of stimulus material.

#### 3.1.2. Stimulus Arousal

“Stimulus arousal” refers to the intensity of metabolic and neural activations of the independent or coactive appetitive or aversive system [77]. Arousal, combined with emotional valence and dominance, has been suggested as a universal, three-dimensional conceptualization of the emotional stimuli [78] in which arousal and valence are culture-free, accounting for major proportion variance in emotional judgment [79, 80]. Reasonably, then, a fair comparison of different expressions requires similar fundamental parameters used in different stimuli. We have found controversial results in previous studies using faces with different emotional valences (i.e., negative and positive biases). Thus, we suggest that stimulus arousal may, in part, be considered responsible for these past results.

A recent meta-analysis of attention studies found a larger negative bias effect for high-arousal scenic or verbal emotional stimuli than for low-arousal stimuli [21]. Although this meta-analysis did not include the factor of face stimuli, other studies have suggested that the degree of arousal also affects the processing of different expressions [81]. For example, in the study by Lundqvist et al. [81], the authors reanalyzed their previous studies (e.g., [16, 82, 83]) and found that the degree of arousal from a picture was highly correlated with the participants' response as the direction of their corresponding superiority effect. At the same time, the researchers asked the participants to rescore the degree of arousal to the photographic stimuli widely used in the visual search research, and they predicted attentional bias based on the arousal score collected from the original stimulus set. The predicted result ultimately fit well with previous studies [81]. Thus, these findings suggest that the contradiction between negative and positive bias in the visual search paradigm is based on the degree of arousal in response to picture stimulation.

No VWM studies have directly investigated the effect of emotional arousal on memory bias toward positive or negative faces. However, although lacking a direct comparison to emotional arousal between happy and angry faces, one study found that different intensities of angry expressions evoked different CDA amplitudes [61]. Specifically, full expressions had a higher amplitude than both subtle (intermediate intensity angry face, morphed from the continuum between neutral and intense angry face) and neutral expressions, while neutral faces had a higher amplitude than subtle expressions, suggesting that different intensities of emotional faces may affect VWM [61]. Studies have also suggested a reduced overall working memory performance when people need to memorize several high-arousal stimuli simultaneously [84]. Taken together, these results indicate that arousal could at least partly affect VWM performance. However, not all previous studies have measured and controlled for a stimulus's arousal level (see Supplementary Table 1; e.g., [55, 56]), and variations exist in the definition of arousal across different studies, i.e., some studies used intensity as their index (e.g., [34]) while others used arousal (e.g., [58]).

In brief, the choice of stimulus material, as well as stimulus arousal, affects the results of both the visual search and the change detection paradigms. However, some studies have used similar materials and obtained different results (e.g., both used schematic faces or photographs but obtained different results), suggesting that differences in stimulus material choices are not the only reason for the inconsistent results. Thus, differences in experimental settings can also account for some variance in results. We further discuss this issue below.

### 3.2. Differences in Experimental Settings

The visual search and change detection are different paradigms; however, several aspects in the experimental settings are similar and affect the experimental results for both paradigms. We next discuss the possible experimental settings that may affect the results of the emotional bias from three main perspectives.

#### 3.2.1. Visual Display Size and Corresponding Time

In both the visual search and change detection paradigms, the visual display set size is an essential index concerning behavioral results, such as the search slope (the function of RT and display set size) in the visual search paradigm and number of VWM representations in the change detection paradigm. Thus, both the display set size and the amount of time given to participants to process the task matter.

Previous attention studies have shown that varying the time settings can lead to differences in the composition of an individual's attention [85]. Using an attention task, researchers have found that a probed stimulus presentation time of 100 ms accompanies an attentional bias toward negative stimuli (such as angry faces in an angry–neutral stimuli pair and neutral faces in a neutral–happy stimuli pair), and this trend was reversed when the presentation time was extended to 500 ms [86]. Although this hypothesis may not explain all the previous studies on the visual search paradigm, the time setting seems to affect the results of emotional bias. For example, in studies supporting a negative bias, participants have usually needed to respond in a limited time [15, 42]. However, in studies supporting a positive bias, participants have usually not had specific time limits for their responses. These trials ended when participants pressed a button (e.g., [16, 17]) or when the interval time was much longer than participants needed (e.g., 10 s in [18] or 30 s in [71]).

VWM studies have found more direct evidence supporting the effect of display size and corresponding time. For example, one study found that high perceptual processing competition (e.g., 150 ms exposure time for encoding) revealed an emotional face advantage (i.e., both happy and angry faces had an advantage over neutral faces). By contrast, an angry face advantage emerged when the competition between stimuli was further increased by increasing the stimulus set size [52]. Furthermore, with the same set size of five, a previous study found a VWM performance cost for fearful faces compared to neutral faces, but only with a longer encoding duration (4,000 ms), as it showed no differences with a shorter encoding duration (1,000 ms [34]: Experiment 1 and Experiment 2). Consistently, the advantage of happy faces compared to angry and fearful faces has also been extractable from a long encoding time condition (4,000 ms [34]: Experiment 4a). These results suggest that the emotional bias in VWM may be affected by the set size and stimulus exposure time of memory array. However, we should note that as the processing time of a single stimulus reduces or extends, the VWM representations might risk being confounded with representations of perception or long-term memory.

#### 3.2.2. The Manner of Stimulus Presentation

The visual search is a very context-dependent process; therefore, discussions of targets should not be isolated from those of background stimuli. This concept is also true for the process of the change detection paradigm in which multiple stimuli are usually presented simultaneously, rather than sequentially. Consequently, differences in the manner of the stimulus presentation for the target and the distractor or background stimuli may also contribute to variations in the results on emotional bias.

For example, the presentation of happy and angry faces in the same visual search array could result in different processing speeds for distractors instead of targets [13, 87]. This hypothesis is mainly applicable to situations where opposite emotions are used as the distractors. For example, one study set a homogenous condition in which all stimuli were presented with the same emotional face. The authors found that participants responded more slowly to all-negative faces than to all-positive and neutral faces [13]. From this point of view, the faster processing of angry target stimuli can be explained by the faster processing of happy distractor stimuli, whereas the slower perception of happy target stimuli can be explained by the degree to which negative faces cause attentional difficulties in attention disengagement from the distractors. Thus, the different setting in distractors may ultimately result in processing differences for both types of target stimuli. In addition, the use of heterogeneous or homogenous identities as a background can also lead to different results. For example, while previous schematic faces had yielded more consistent results for a negative bias, a positive bias emerged when a heterogeneous background was used [50]. However, this phenomenon does not fully explain the results obtained with photographs because some studies with a heterogeneous background showed a positive bias [16, 18], while others showed a negative bias [40, 45].

The effect of the manner of presentation may be generalized to the findings of VWM studies. Previous studies can be roughly divided into two kinds of settings in terms of stimulus presentation, namely, different identities with the same expression [27, 28] and the same identity with a different expression [52, 56]. Although these settings do not appear to directly cause different results, differences in stimulus presentation have occurred across studies despite the use of a similar experimental paradigm. In addition, the change detection paradigm typically involves two stimulus arrays, a “memory array” and a “probe array.” The patterns of both arrays affect the experimental results, and the results may also be influenced by the visual search process itself—either at the memory array or the probe array. Besides memory maintenance, memory filtering is another essential aspect of studying VWM. The manipulation of fearful and neutral faces as targets or distractors in a change detection task has revealed in previous studies that—in general—fearful faces are more challenging to filter than are neutral faces, thereby reflecting a larger CDA amplitude in the fearful-distractor-with-neutral-target condition [54]. Follow-up behavioral and fMRI studies found similar result patterns [88, 89]. Ye et al. [90], who used the CDA component, found that participants with high VWM capacity were able to filter all the facial distractors from VWM, regardless of their expression, while low-capacity participants failed to filter the neutral and angry faces but efficiently filtered happy faces. In addition, a follow-up study used a similar paradigm and found that participants in the personal relative deprivation group failed to filter out neutral or angry facial distractors but succeeded in filtering out happy facial distractors from VWM [91]. All these studies suggest that the expression types of stimuli modulate both storage and distractor filtering in VWM. From this point of view, the use of the same or different emotional faces in a memory array could also lead to different results.

#### 3.2.3. Differing Demands in Experiments

Another important aspect in experimental settings relates to the observers. We human beings, as subjective animals with our own thoughts, may also be indirectly affected by how experimenters provide instructions and by our own understanding of an experiment. As Supplementary Table 1 shows, although the paradigm remains basically the same, the participants' task can be further divided (e.g., detect whether identity is present or absent, detect whether identity is the same or different, detect whether the expression is the same or different, and detect whether the probe is the same or different). Therefore, the demands placed by the experiment and the participants' own strategies in understanding the task instructions could partially affect the results of emotional bias.

Previous studies using a visual search have suggested employing a fixed target to avoid the discrepancies caused by different strategies across participants. That is, the specific target would be given an emotion (e.g., happy face) at the beginning of the task, and the participants were then asked to constantly search for this target emotion across trials [35]. Although this type of control reduces the variation in subjects' own search strategies, we argue that it also makes the search task more difficult to distinguish from the recognition task. Unlike the controversial results on the visual search, which require a rapid but less in-depth process, expression recognition studies have more consistently supported positive bias [37]. Most of the previous visual search studies supporting negative bias also did not specify the target stimulus before conducting their experiments with participants [12, 13, 43]. On the contrary, studies in favor of positive bias have often asked participants to find target stimuli for specific emotions (i.e., they used a fixed target [16, 18, 47]). These results also raise concerns that some of the positive bias findings might be confounded with the interference of face recognition.

A similar impact from experiment instruction can also occur in VWM studies using the change detection paradigm. For example, the information that participants were required to remember has differed across studies (see column 8 in Supplementary Table 1). Some studies have regarded emotional information as a form of task-independent information [27, 28, 30, 34], while others have regarded the expression as task-related information [31, 32, 52]. Although this setup difference may not directly explain the observed discrepancy, a deeper processing of emotional information seems to be more likely to trigger positive bias. For example, in a relocated task [33], or when a longer encoding time was provided [34], the happy face advantage emerged in VWM.

These results suggest that different experimental settings may involve different cognitive resources. Therefore, by moving beyond these methodological challenges, a more likely explanation for the conflicting results of previous studies is that negative bias and positive bias act at different cognitive stages.

### 3.3. Different Stages in the Cognitive Process

In both the visual search and the change detection paradigms, the participants must finish several cognitive processes to accomplish their whole task. In attention research, the process of the visual search paradigm has, conventionally, contained at least two distinct but interrelated stages: the preattentive stage and the attentive or postattentive stage. The preattentive stage occurs before the attentional selection of a target stimulus. In this stage, the process does not require attentional allocation to the stimulus, whereas the attentive or postattentive process involves the direct focus on a target stimulus [92]. Calvo et al. [48], who used eye movement techniques, proposed a third stage of visual search for emotional faces called “decision efficiency.” The decision efficiency stage occurs immediately before decision-making, as the varying decision times between fixing the gaze on the target stimulus and making a choice have shown for different emotional faces [48]. For VWM studies, the change detection paradigm process comprises four stages: the encoding stage, the consolidation stage, the maintenance stage, and the retrieval stage [93]. The encoding stage in VWM overlaps with the processes in attention research, during which, perception representations are created and then consolidated into VWM representations during the consolidation stage. After the stimulus disappears, the participants need to “maintain” VWM representations and then “retrieve” them in subsequent tasks to complete the whole cognitive process of VWM. In addition, the VWM consolidation comprises two different stages [94–96]. In the early consolidation stage, individuals automatically create low-precision representations. Subsequently, in the late consolidation stage, individuals can voluntarily create high-precision representations.

For visual search studies, one possibility is that an automatic bias toward negative emotions exists in the early preattentive stage, whereas the positive bias is revealed in the later recognition and/or decision-making stages. Consistent with this point of view, the use of an emotion classification task combined with the EEG technique has revealed that N170, in the early stage, showed a higher response to negative faces—such as angry, fearful, and sad faces. By contrast, happy faces tended to correlate with facilitation in categorization (reflected by P3b) and decision-making (reflected by a slow positive wave in the later stage) [97]. LeDoux [98] concluded from animal model studies that the fear response could comprise two pathways. In the subcortical pathway, information is sent rapidly and directly to the amygdala. By contrast, in the cortical path, information is sent to the cortex for subsequent analysis before reaching the amygdala. Therefore, the subcortical pathway activates the amygdala in advance and enables a ready state for fearful information. Thus, once information on the cortical path is transmitted to the amygdala, the individual can respond immediately. Therefore, the amygdala can combine limited information for a rough but rapid assessment of threat stimulation at the early stage. This first stage of quick evaluation is likely the neural mechanism that produces the superiority effect of threat stimuli (angry and fearful faces). However, other emotional information (i.e., a happy face) may reach the cortical path with more comprehensive processing. Studies have confirmed that although happy faces can also activate the amygdala, the effect is mainly observed at the later stimulus presentation time [99]. On the contrary, Becker and Rheem [36] have an opposite view and suggest that threatening faces are privileged at a later stage because of the difficulty of attention disengagement. For either order, however, future studies will need to separate the different stages, as this may help to shed light on the real reasons for the discrepancies in previous results.

Similarly, for VWM studies, although memory usually requires more in-depth processing of task-related information, different emotional information could also affect VWM at different processing stages. For example, different expressions did not show any effect at the encoding stage, but emotional faces (both angry and happy) showed a greater resource allocation at the maintenance stage [58]. Information with different emotional valences also influences VWM via different neural bases [100]. More importantly, previous studies have not been able to dissociate attention from VWM. Therefore, whether attention or VWM is responsible for this discrepancy is difficult to discern.

In conclusion, after controlling for the effects of stimulus materials and experimental procedures, further delineation of different cognitive processing stages may be an effective way to resolve previous conflicts.

## 4. Summary and Prospects

In this paper, we have mainly considered studies on attention and VWM using different emotional faces, and we have proposed three possible factors that could explain the mixed results of the previous studies. A recent study by Becker and Rheem [36] listed five necessary points of guidance for future researchers who use the visual search paradigm to study expressions. (Extracted from the conclusion of Searching for a Face in the Crowd: Pitfalls and Unexplored Possibilities ([36], p. 635). “(a) Vary the crowd size so that search slopes can be assessed. (b) Account for the speed with which distractors are rejected by considering the target-absent search rates or ensure that all of the distractor arrays are equivalent. (c) Ensure that participants are processing the stimulus signal of interest rather than low-level features that are correlated with this signal. (d) Vary the distractors and targets in ways that keep participants from learning to use any low-level features to complete the task. (e) Jitter the positions of the items in the crowds so that textural gestalts cannot be exploited.”) In addition to their guidance, we offer several other suggestions for addressing the problems common to both the visual search paradigm and the change detection paradigm. We first discuss the limitations and recommendations of the existing paradigms related to the visual search and change detection paradigms in order to minimize discrepancies. We then propose some possible directions for future research.

### 4.1. The Choice of Emotional Stimuli

Above all, in studies of change detection and visual search, researchers need to be more careful in the selection of stimulus materials, especially regarding the control of low-level physical features and stimuli's arousal. The degree of arousal resulting from the stimulus itself should be defined (e.g., distinguish between arousal and intensity) and evaluated comprehensively. Collecting the participants' own arousal evaluations for each experimental stimulus within the study is also important since arousal as such is subjective. We offer three other suggestions for the selection of emotional stimuli.

First, future research should pay more attention to the selection of photographs and schematic faces. Therefore, more advanced technology for further control of facial expression—for example, using computer-generated techniques to create human-like pictures [101]—is needed in future work. The application of dynamic facial expressions, as well as body expressions, also offers possible directions for future exploration [40, 102, 103].

First, future research should pay more attention to the selection of photographs and schematic faces in terms of physical features. Therefore, more advanced technology or accurate way for further control of physical features—for example, using computer-generated techniques to create human-like pictures [101]—is needed in future work. In addition, the application of dynamic facial expressions, as well as body expressions, also offers possible directions that future research should explore [18, 102, 103].

Second, both attention and VWM studies have considered the use of neutral faces as a baseline setting for comparison with emotional faces. However, neutral faces are more likely to be perceived as negative than positive [19]. This tendency may lead to imbalance in a search array or the encoding stage of a memory array. The use of fearful and angry faces for the threat effect should also be interpreted with caution. Although fearful and angry faces have usually been classified into the same category as threatening faces by previous studies (e.g., [13, 42, 43]), they actually contain different information. The threat source of anger is basically the face per se, while fear serves as a reminder of the threat in the viewer's environment [34, 104]. Therefore, future studies should discuss fearful and angry faces separately, rather than simply categorizing both of them as threatening stimuli.

Third, since emotional faces (e.g., angry faces) are already a source of emotional information per se, another question that future studies should address is whether the currently available results are due to emotional states triggered by expression stimuli. The answer to this question may be negative, mainly because emotional induction usually takes time and needs to remain relatively stable. In typical visual search and change detection paradigms, different emotional faces (positive and negative) often randomly appear in the same trial or in adjacent trials, which can create difficulty for the participants to form a stable emotional state. Thus, emotional states should not be the main cause of the previous controversial studies. However, this suggestion does not negate the effects of emotional states on an individual's processing of attentional or memory tasks. Indeed, previous studies have shown that emotional states or mental illnesses (e.g., depression, anxiety, and worry) can affect attention and VWM [64, 65, 88, 105–109]. However, knowledge is currently limited regarding the influence of emotional states on the results of the visual search or change detection paradigms that use emotional face stimuli. This area should therefore be explored further in future research.

### 4.2. Standardization of the Experimental Setting

Based on our summary, the experimental settings for both paradigms evidently require further standardization. For example, when testing different visual matrix sizes, future studies should also consider the timing of the stimulus presentation and explore the effects of different combinations of stimulus set sizes and times for both paradigms. The experimental instructions should also be carefully controlled to prevent the involvement of unnecessary cognitive processes.

Most previous studies have used the visual search paradigm and change detection paradigm to investigate emotional face processing in attention and VWM; however, some other paradigms can investigate similar topics in these fields. For example, in the field of attention, the dot-probe paradigm [86], rapid serial visual presentation task (RSVP) [110], and visual crowding paradigm [111] can also explore attentional bias to emotional faces. Similar contradictory results have also been found for emotional bias in studies using the RSVP paradigm (for negative bias, see [112]; for positive bias, see [113]). Some studies have even suggested that VWM and the attentional blink observed in the RSVP paradigm might share the same neural processing and storage capacity mechanisms [52, 114]. In the VWM field, the N-back task [115] is also an appropriate paradigm for testing emotional bias. A growing body of research has used N-back tasks or other tasks to explore the potential differential impact of emotional faces versus neutral faces (for a review, see [116]). Thus, future research should examine whether paradigm types modulate emotional bias in attention and VWM. Likewise, many of the issues mentioned in this paper (e.g., selection of stimulus materials) are applicable to other attention or VWM studies.

### 4.3. Controlling and Tracking Cognitive Processes

Future studies also need to explore the causes of the positive and negative biases underlying different cognitive processes. This exploration will require that future studies define and divide the different processing stages in corresponding paradigms. Future studies can succeed in this regard by combining traditional behavioral indicators with other neuroscience techniques. Specifically, they can combine different ERP indicators (e.g., N2pc in visual attention studies and CDA in VWM studies) or combine EEG with eye movements to generate fixation-based ERPs [117].

In VWM studies, both attention and memory play vital roles; therefore, different emotional advantages may already exist in the attention process rather than in the memory process. This makes determining whether attention or memory processes caused the mixed results from VWM studies in emotional advantages rather difficult. Future studies can try to separate the attention-related process from the VWM-related process when exploring emotional face advantages in VWM. Alternatively, future studies could include attention and VWM in the same context (e.g., using similar stimuli and experimental settings) and examine the associations between visual attention and VWM. For example, previous study showed a high correlation between the reciprocals of VWM capacity and the visual search slope with line-drawing objects [118]. Therefore, a joint study of these two paradigms could be a feasible alternative to better study the role that attention serves in the emotional bias of VWM.

## 5. Conclusion

This review of the literature supports the view that the mixed results from previous studies could have been arisen due to differences in stimuli, experimental settings, and processing stages at the neural level. The empirical research and the theoretical background indicate that both negative and positive biases are likely. However, if we eliminate the influence of the stimulus materials and experimental settings, a more likely explanation would be that both biases occur but in different cognitive stages. Researchers should adapt more comparable and well-designed paradigms to provide new evidence of positive and negative bias for emotional faces in future studies. A combination of neuroscience techniques and advanced data analysis should be also applied to this field to provide a better understanding of the mechanism behind the advantage effect of different expressions. We believe that the adoption of these suggestions will help to settle the controversy of positive/negative emotional bias in visual attention and VWM.

## Supplementary Materials

Supplementary materials Supplementary Material 1 summarizes the paradigms and measures used in visual search and change detection paradigm. Supplementary Table 1 lists the key setups and results in previous VWM studies, especially on the adoption of the change detection paradigm.
