# Supplementary material for: Negative and Positive Bias for Emotional Faces: Evidence from the Attention and Working Memory Paradigms
Source: Neural Plast. 2021 May 27;2021:8851066. doi: 10.1155/2021/8851066 (PMC8178010; doi:10.1155/2021/8851066)
Supplement: Supplementary materials — Supplementary Material 1 summarizes the paradigms and measures used in visual search and change detection paradigm. Supplementary Table 1 lists the key setups and results in previous VWM studies, especially on the adoption of the change detection paradigm. [file 8851066.f1.pdf]

Supplementary Material

Negative and Positive Bias for Emotional Faces: Evidence from the Attention and Working Memory Paradigms

Qianru Xu[1,2], Chaoxiong Ye[1,2]*, Simeng Gu[1,3], Zhonghua Hu[1], Yi Lei[1], Xueyan Li[4], Lihui Huang[5], Qiang Liu[1]

1 Institute of Brain and Psychological Sciences, Sichuan Normal University, Chengdu, China;
2 Department of Psychology, University of Jyvaskyla, Jyväskylä, Finland;
3 Department of Medical Psychology, Medical School in Jiangsu University, Zhenjiang, China;
4 School of Foreign Languages, Dalian University of Technology, Dalian, China;
5 Faculty of Education, Sichuan Normal University, Chengdu, China;

*** Correspondence to:**

Chaoxiong Ye, Ph.D.

Institute of Brain and Psychological Sciences,

Sichuan Normal University, Chengdu, 610000, China

Email: cxye1988@163.com

Paradigms and Measures Used in Visual Search and Change Detection Paradigm

The visual search paradigm (also called the "face in the crowd paradigm" when using faces as stimuli) mimics the process of detecting target objects or people from multifarious information of everyday lives [1,2]. In a classic visual search paradigm experiment (see Figure 1 in the main text), each trial contained a face array, and half of the trials contained a target stimulus (i.e., one face showed different expressions than the other faces) while the other half of the trials did not contain a target stimulus (i.e., all the faces showed the same expression). Participants are asked to detect whether a face presented different expressions from the other faces. For the analysis of results, the reaction time (RT), accuracy rate (ACC), error rate, and search slope (the function of RT and set size) are behavioral indices to evaluate participants' efficiency in detecting the search target. The shallower the search slope of a target stimulus, the more efficient the search. Moreover, the search slope can also indicate whether the processing method uses a parallel or serial search approach [2]. In addition to the behavioral index, Luck and Hillyard [3] found an event-related potentials (ERPs) component called N2-posterior-contralateral (N2pc) which has been commonly used by recent studies as an indicator to track the visual selective attention process. N2pc is mainly distributed in the posterior-contralateral electrodes, appearing 200–300 ms after stimulus presentation (for a review, see [4]). The latency of the N2pc indicates the time point at which attention focused on the target item, while amplitude can reflect the amount of attention on the target [4,5]. Additionally, several other ERP components—such as N170, early posterior negativity (EPN), and late positive potential (LPP)—have usually been used to indicate emotional processing in visual attention tasks (for a review, see [6]).

VWM research on emotional bias has widely used the change detection paradigm. This paradigm (also called the "visual short-term memory task" [see [7]] or the "match-to-sample probe recognition task" [see [8]]) generally comprises four parts: pre-stimulus fixation, memory array, retention interval, and probe array (see Figure 2 in the main text). Typically, in half of the related trials, the probe array and memory array are exactly the same, and in the other half of the trials, one of the memory items in the probe array differs from the memory array, and the participant's task is to detect whether or not a change has occurred. Generally, the change detection paradigm asks participants to temporarily store the memory array across a blank delay period and then compare the memory array with the probe array. Accuracy, Cowan's K[1] [9], and *d'*[2] [10], are common behavioral indicators in the change detection paradigm. Cowan's K is often used to measure individuals' VWM capacity. It is an estimated index of the number of items successfully stored in an individual's VWM. Meanwhile, *d'* is an index of VWM performance. It is obtained from the signal detection theory, representing the distance between the z-transforms of the hit rate and false alarm rate [10]. Also, researchers have identified an ERP component called

---

[1] K = *set size* × (hit rate – false alarm rate).

[2] *d'* = Z(hit rate) – Z(false alarm rate).

“contralateral delay activity” (CDA, also known as “sustained posterior contralateral negativity,” SPCN; [11]) as a neural indicator of VWM maintenance. The CDA’s amplitude increases with the number of items in an individual’s VWM during the maintenance of memory material, and it reaches an asymptote once approximately three to four simple objects have been stored, reflecting the limitation of an individual’s VWM capacity [12]. In addition, N170, N250 (which usually reflects the repetition effect) and P3b (the subcomponent of P300, which is sensitive to the resource allocation process) have also been suggested to relate with VWM emotional processing (e.g., [13,14]).

Supplementary Table 1: Studies on emotional face in visual working memory (VWM) by using change detection paradigm.

Study [a]	N	Expression	Stimulus type/ Stimulus set [b]	Stimulus arousal	Set size	Encoding time (ms)	Task [d]	Behavior /Neural index	Results [e]	Comments
Becker et al. (2014)[15]: Exp. 1	64	Neutral, angry	Photograph /- [c]	-	4	10000	Detect identity present/ absent	Hit rate, false alarm rate, *d'*, bias (c)	Angry > neutral	Test gender effect
Brenner et al. (2014)[16]	29	Positive (very happy, somewhat happy), neutral, Negative (sad, fearful, and angry)	Photograph /NimStim	-	1	200	Detect expression same/ different	Acc, RT, P100, N170, N250, theta power	Negative > positive	
Curby et al. (2019)[8]: Exp. 1	40	Fearful, neutral	Photograph /NimStim, KDEF, CVL	-	5	1000/4000	Detect probe same/ different	K	1000 ms: no effect; 4000 ms: fearful < neutral	
Curby et al. (2019)[8]: Exp. 2	41	Fearful, neutral	Photograph /NimStim, KDEF, CVL	-	5	1000/4000	Detect probe same/ different	K	1000 ms: no effect; 4000 ms: fearful < neutral	Also test inverted face
Curby et al. (2019)[8]: Exp. 3	82	Fearful, neutral	Photograph /NimStim, KDEF, CVL	-	2,4,6	4000	Detect probe same/ different	K	Fearful < neutral	Test emotional state, and attentional control

Study [a]	N	Expression	Stimulus type/ Stimulus set [b]	Stimulus arousal	Set size	Encoding time (ms)	Task [d]	Behavior /Neural index	Results [e]	Comments
Curby et al. (2019)[8]: Exp. 4a	42	Neutral, happy, fearful, angry	Photograph /Radboud	Happy = fearful; fearful ≠ angry; happy ≠ angry	5	4000	Detect probe same/ different	K	Fearful, angry < happy	
Jackson et al. (2008)[17]	35	Angry, happy, neutral	Photograph /Ekman & Friesen	Happy = angry	1,2,3,4	2000	Detect identity present/ absent	*d'*, fMRI	Angry> happy; angry> neutral	Emotion effect showed on rSTS, rPFC (IFS), and rGPi
Jackson et al. (2009)[7]: Exp. 1	24	Angry, happy, neutral	Photograph /Ekman & Friesen	-	1,2,3,4	2000	Detect identity present/ absent	*d'*, K-iterative (K_{it})	Angry > happy; angry > neutral	
Jackson et al. (2009)[7]: Exp. 2	18	Angry, happy, neutral	Photograph /Ekman & Friesen	Angry = happy	2,4	2000	Detect identity present/ absent	*d'*, K-iterative (K_{it})	Angry > happy; angry > neutral	High or low-arousal music was played during the experiment
Jackson et al. (2009)[7]: Exp. 3	26	Angry, neutral	Photograph /Ekman & Friesen	-	2,4	4000	Detect identity present/ absent	*d'*, K-iterative (K_{it})	Angry > neutral	
Jackson et al. (2009)[7]: Exp. 5	40	Angry, happy, neutral	Photograph /Ekman & Friesen, KDEF	Morphed to the same intensity	2,4	2000	Detect identity present/ absent	*d'*, K-iterative (K_{it})	Angry > happy; angry > neutral	16 participants performed with Ekman face set, 24 performed with KDEF face set

Study [a]	N	Expression	Stimulus type/ Stimulus set [b]	Stimulus arousal	Set size	Encoding time (ms)	Task [d]	Behavior /Neural index	Results [e]	Comments
Jackson et al. (2012)[18]: Exp. 1	25	Angry, happy	Photograph /Ekman & Friesen	-	2	2000	Detect identity present/ absent	*d'*, RT	Angry = happy	Intervening neutral words vs. no words during maintenance
Jackson et al. (2012)[18]: Exp. 2	27	Angry, happy	Photograph /Ekman & Friesen	-	2	2000	Detect identity present/ absent	*d'*, RT	Angry face was boosted by intervened emotional word, but happy face was not affected by it	Intervening negative, positive, or neutral words during maintenance
Jackson et al. (2014)[19]: Exp. 1	22	Angry, happy	Photograph /Ekman & Friesen	Angry= happy	1,2,3,4	2000	Detect identity present/ absent	Hits, false alarm rate, *d'*, RT	Angry > happy	Emotional faces presented at encoding phase.
Jackson et al. (2014)[19]: Exp. 2	13	Angry, happy	Photograph /Ekman & Friesen	Angry= happy	1,2,3,4	2000	Detect identity present/ absent	Hits, false alarm rate, *d'*, RT	Angry = happy	Emotional faces presented at retrieval phase
Jackson et al. (2014)[19]: Exp. 3	25	Angry, happy	Photograph /Ekman & Friesen	Angry= happy	2	2000	Detect identity present/ absent	Hits, false alarm rate, *d'*, RT	No effect of word valence for happy faces, but negative intervening word boosted angry face WM performance	Intervening a word valence categorization during maintenance
Langeslag et al. (2009)[13]	29	Angry, happy, neutral	Photograph /Ekman & Friesen	Angry= happy	1,3	2000	Detect identity present/ absent	Pr, Br, RT, P1, N170, P3b, N250r	Angry > neutral; happy > neutral	

Study [a]	N	Expression	Stimulus type/ Stimulus set [b]	Stimulus arousal	Set size	Encoding time (ms)	Task [d]	Behavior /Neural index	Results [e]	Comments
Lee and Cho (2019)[20]: Exp. 1	32	Fearful, happy, neutral	Photograph /KUFEC	Feaful: 4.2 Happy: 4.46	4	1200	Detect identity same/ different	*d'*	Fearful, neutral group > happy, neutral group; fearful > neutral; happy > neutral	Half of participants performed fearful and neutral faces, while half performed happy and neutral faces
Lee and Cho (2019)[20]: Exp. 2	32	Fearful, happy, neutral	Photograph /KUFEC	Feaful: 4.2 Happy: 4.46	4	1200 (300 ms for each face)	Detect identity same/ different	*d'*	Fearful, neutral group > happy, neutral group; fearful > neutral	Memory array stimuli presented sequentially
Lee and Cho (2019)[20]: Exp. 3	32	Fearful, happy, neutral	Photograph /KUFEC	Feaful: 4.2 Happy: 4.46	4	1200 (300 ms for each face)	Detect identity same/ different	*d'*	Fearful, neutral group = happy, neutral group; fearful > neutral	Memory array stimuli presented sequentially and a blank interval of 300 ms appeared between images

**Study [a]**	**N**	**Expression**	**Stimulus type/ Stimulus set [b]**	**Stimulus arousal**	**Set size**	**Encoding time (ms)**	**Task [d]**	**Behavior /Neural index**	**Results [e]**	**Comments**
Linden et al. (2011)[21]: Exp. 1	20	Angry, happy, neutral, sad, fearful	Photograph /Ekman & Friesen	Angry, fearful higher than all other condition, sad & happy > neutral	2	2000	Detect identity present/ absent	*d'*	Angry > fearful; happy > fearful; neutral > fearful	Also test 20 melancholiac depression and 20 non-melancholic depression participants, and sad face benefits showed in melancholic group
Liu et al. (2020)[14]	38	Happy, sad	Photograph /Chinese facial expression of emotion system	Happy = sad	2	2000	Detect identity present/ absent	Hits, CR, FA, RTs, *d'* N170, VPP, N250, P3b, LPP	Happy > sad	

Study [a]	N	Expression	Stimulus type/ Stimulus set [b]	Stimulus arousal	Set size	Encoding time (ms)	Task [d]	Behavior /Neural index	Results [e]	Comments
Maran et al. (2015)[22]: Exp.1	24	Angry, happy, anxious, neutral	Photograph /NimStim	No difference in perceived intensity	4	2000	Detect identity present/ absent	*d'*	Neutral arousing condition: angry > happy; angry > anxious; angry > neutral; happy > anxious. Negative arousing condition: equalized VWM performance for all expression	Under unpleasant/ neutral affect emotional state

Study [a]	N	Expression	Stimulus type/ Stimulus set [b]	Stimulus arousal	Set size	Encoding time (ms)	Task [d]	Behavior /Neural index	Results [e]	Comments
Maran et al. (2015)[22]: Exp.2	30	Angry, happy, anxious, neutral	Photograph /NimStim	No difference in perceived intensity	4	2000	Detect identity present/ absent	*d'*	Neutral arousing condition: angry > happy; angry > anxious; angry > neutral. Positive arousing condition: equalized VWM performance for all expression	Under pleasant/neutral affect emotional state
Sessa et al. (2011)[23]	28	Fearful, neutral	Photograph /Ekman & Friesen, KDEF	-	2,4	200	Detect identity same/ different	*d'*, K, K_{it}, RT, CDA	Fearful > neutral	
Sessa et al. (2018)[24]	29	Neutral, subtle angry, full angry	Photograph /KDEF	Morphed from neutral to full angry	2	500	Detect expression same/ different	Mean proportion of correct responses, CDA	Full angry > neutral > subtle angry	
Simione et al. (2014)[25]: Exp. 1	19	Angry, happy, neutral	Schematic face	-	4	150/400	Report the expression at the probed location	Acc	Emotion > neutral, but no difference between angry and happy	

Study [a]	N	Expression	Stimulus type/ Stimulus set [b]	Stimulus arousal	Set size	Encoding time (ms)	Task [d]	Behavior /Neural index	Results [e]	Comments
Simione et al. (2014)[25]: Exp. 2	20	Angry, happy, neutral	Schematic face	-	3,5	150	Report the expression at the probed location	Acc	Angry > neutral; angry > happy	
Spotorno et al.(2018)[26]: Exp. 1	48	Angry, happy	Photograph /Radboud	-	1,2,3,4	1500 ×set size	Drag the face to the original location	Acc, precision, swap error, oculomotor activity	Happy > angry	
Spotorno et al.(2018)[26]: Exp. 2	48	Angry, happy	Photograph /Radboud database	-	4	6000	Drag the face to the original location	Acc, precision, swap error, oculomotor activity	Happy > angry	Test the effect of the maintenance time
Švegar et al. (2013)[27]	24	Afraid, angry, disgusted, happy, neutral, sad, surprised	Photograph /AKDEF, KDEF	-	6	2000	Detect expression same/different	Percentage of correct answers; RT	Happy > all the other emotions while no difference among other emotions	
Thomas et al. (2014)[28]: Exp. 1	25	Angry, happy, neutral	Photograph /Ekman & Friesen	-	4	2000	Detect identity present/absent	*d'*, RT	Angry > happy	Compare the uniform memory array and singleton memory array

**Study [a]**	**N**	**Expression**	**Stimulus type/ Stimulus set [b]**	**Stimulus arousal**	**Set size**	**Encoding time (ms)**	**Task [d]**	**Behavior /Neural index**	**Results [e]**	**Comments**
Thomas et al. (2014)[28]: Exp. 2	32	Angry, happy, neutral	Photograph /Ekman & Friesen	-	4	2000	Detect identity present/ absent	*d'*, RT, eye movement	Angry > happy	The procedure was same as the singleton condition in Exp.1, but they also monitored the eye movement here and verbal suppression was omitted.
Xie et al. (2017)[29]: Exp. 1	60	Positive set (5 happy + 1 neutral); negative set (5 sad + 1 neutral)	Schematic face	Positive = negative	4	500	Localize the changed face	K	Positive = negative; K (neutral color)> K (emotional face); higher WM capacity maintain more happy faces	WM capacity measured by color

Study [a]	N	Expression	Stimulus type/ Stimulus set [b]	Stimulus arousal	Set size	Encoding time (ms)	Task [d]	Behavior /Neural index	Results [e]	Comments
Xie et al. (2017)[29]: Exp. 2	42	Positive set (5 happy + 1 neutral); negative set (5 sad + 1 neutral)	Schematic face	Positive = negative	4	500	Localize the changed face	K	Positive = negative; K (emotional face)> K (neutral line drawing symbols); higher WM capacity maintain more happy faces	WM capacity measured by line-drawing symbols
Zhou et al. (2021)[30]	31	Happy, sad	Photograph /Chinese facial expression of emotion system	Happy = sad	2	2000	Detect identity present/absent	Hits, CR, FA, RTs, *d'* N170, VPP, P3b, LPP	Happy = sad	Depressed group: sad > happy

Abbreviations:
Column 1 (Study): Exp., Experiment
Column 2 (N): N, participants numbers
Column 9 (Behavior/Neural index): Acc, accuracy; RT, reaction time; d', VWM performance index, d' = Z(hit rate) – Z(false alarm rate); K, VWM capacity index, K= set size × (hit rate – false alarm rate); fMRI, functional magnetic resonance imaging; K-iterative (Kit), calculated by an iterative procedure, please see details in Jackson et al. [7]; Pr, discrimination index, Pr = hit rate – false alarm rate; Br, response bias index, Br = false alarm rate/(1-Pr), Please see more details for Pr and Br in Langeslag et al. [13]; CR, correct rejections; CDA, contralateral delay activity, also known as sustained posterior contralateral negativity, SPCN, neural index of VWM maintenance.
Column 11 (Comments): rSTS, right superior temporal sulcus; rPFC, right prefrontal cortex; IFS, inferior frontal sulcus; rGPi, right globus pallidus internus
a. The missing experiment here is due to the sub-experiment unrelated to the change detection paradigm or the main topic of this review, e.g., Experiment 2 in Becker et al. [15] is more related to race than emotional processing; Experiment 4 in Jackson et al. [7] is a discrimination task; Experiment 2 and 3 in Linden et al. [21] are emotional classification task and arousal/valance rating task, respectively.
b. We listed here both the stimulus material types (photograph or schematic face) and the stimulus set of photographs for a better comparison between different stimulus databases. The main database used in previous studies included: Ekman & Friesen set [31]; KDEF, Karolinska Directed Emotional Faces database

[32], AKDEF, Averaged Karolinska Directed Emotional Faces [33]; NimStim database [34]; CVL Face Database [35]; Radboud Face Database [36]; KUFEC, Korea University Facial Expression Collection [37]; Chinese facial expression of emotion system [38].
c. "-" means the information was not reported in the relevant publications.
d. Here we classified the tasks into four types based on what the researchers reported in relevant studies: 1) detect identity present/absent, 2) detect identity same/different, 3) detect expression same/different; 4) detect probe same/different. "Present/absent" refers to studies using multiple stimuli in the encoding array, with only one single stimulus in the central of the probe array, and the main task of the participant was to report whether or not the single probe was presented in the memory array; "Same/different" refers to studies using multiple stimuli (or single stimulus in the probe array but the face present in one of the locations from the study array rather than central space) both in the memory array and the probe array, and participants needed to indicate whether the probe array was identical to the memory array or whether one of the faces had changed. "Detect identity" refers to the expression irrelative to the study, and participants only needed to detect whether the identity had changed, whereas "Detect expression" meant that the participants needed to detect whether the expression on the probe array was identical to the memory array while the identity stayed unchanged or needed to be ignored. Additionally, "Detect probe" refers to the relevant study that did not emphasize detecting the expression or the identity but required participants to determine whether the whole probe face (both identity and expression) was identical to the memory items. In addition, a few studies have used a similar but not the classical change detection task, we decided to include those studies here for a better comparison. For those studies, we have only presented the specific tasks reported in the relevant studies without placing them in any of the above categories.
e. Only the main and most consistent results are reported here. " > " refers to the bias toward the former expression compared to the latter one; " < " refers to the bias toward the latter expression compared to the former one; " = " refers to no significant difference between the two expressions.

Reference:

[1] A.M. Treisman, G. Gelade, A feature-integration theory of attention., Cogn. Psychol. 12 (1980) 97–136. https://doi.org/10.1016/0010-0285(80)90005-5.

[2] A. Frischen, J.D. Eastwood, D. Smilek, Visual search for faces with emotional expressions., Psychol. Bull. 134 (2008) 662–676. https://doi.org/10.1037/0033-2909.134.5.662.

[3] S.J. Luck, S.A. Hillyard, Electrophysiological correlates of feature analysis during visual search, Psychophysiology. 31 (1994) 291–308.

[4] S.J. Luck, Electrophysiological Correlates of the Focusing of Attention within Complex Visual Scenes: N2pc and Related ERP Components, Oxford Handb. Event-Related Potential Components. (2012). https://doi.org/10.1093/oxfordhb/9780195374148.013.0161.

[5] T. Feldmann-Wüstefeld, M. Schmidt-Daffy, A. Schubö, Neural evidence for the threat detection advantage: Differential attention allocation to angry and happy faces, Psychophysiology. 48 (2011) 697–707. https://doi.org/10.1111/j.1469-8986.2010.01130.x.

[6] S. Schindler, F. Bublatzky, Attention and emotion: An integrative review of emotional face processing as a function of attention, Cortex. (2020) 1–25. https://doi.org/10.1016/j.cortex.2020.06.010.

[7] M.C. Jackson, C.Y. Wu, D.E.J. Linden, J.E. Raymond, Enhanced Visual Short-Term Memory for Angry Faces, J. Exp. Psychol. Hum. Percept. Perform. 35 (2009) 363–374. https://doi.org/10.1037/a0013895.

[8] K.M. Curby, S.D. Smith, D. Moerel, A. Dyson, The cost of facing fear: Visual working memory is impaired for faces expressing fear, Br. J. Psychol. 110 (2019) 428–448. https://doi.org/10.1111/bjop.12324.

[9] N. Cowan, The magical number 4 in short-term memory: A reconsideration of mental storage capacity Behavioral and Brain Sciences, Behav. Brain Sci. 24 (2001) 87–114.

[10] P. Wilken, W.J. Ma, A detection theory account of change detection, J. Vis. 4 (2004) 1120–1135. https://doi.org/10.1167/4.12.11.

[11] E.K. Vogel, M.G. Machizawa, Neural activity predicts individual differences in visual working memory capacity, Nature. 428 (2004) 748–751. https://doi.org/10.1038/nature02447.

[12] R. Luria, H. Balaban, E. Awh, E.K. Vogel, The contralateral delay activity as a neural measure of visual working memory, Neurosci. Biobehav. Rev. 62 (2016) 100–108. https://doi.org/10.1016/j.neubiorev.2016.01.003.

[13] S.J.E. Langeslag, H.M. Morgan, M.C. Jackson, D.E.J. Linden, J.W. Van Strien, Electrophysiological correlates of improved short-term memory for emotional faces, Neuropsychologia. 47 (2009) 887–896. https://doi.org/10.1016/j.neuropsychologia.2008.12.024.

[14] M. Liu, L. Zhou, X. Wang, B. Ye, Sad expressions during encoding attenuate recognition of facial identity in visual working memory: behavioural and electrophysiological evidence, Cogn. Emot. 0 (2020) 1–13. https://doi.org/10.1080/02699931.2020.1726291.

[15] D.V. Becker, C.R. Mortensen, U.S. Anderson, T. Sasaki, Out of sight but not out of mind: Memory scanning is attuned to threatening faces, Evol. Psychol. 12 (2014) 901–912. https://doi.org/10.1177/147470491401200504.

[16] C.A. Brenner, S.P. Rumak, A.M.N. Burns, P.D. Kieffaber, The role of encoding and attention in facial emotion memory: An EEG investigation, Int. J. Psychophysiol. 93 (2014) 398–410.

https://doi.org/10.1016/j.ijpsycho.2014.06.006.
[17] M.C. Jackson, C. Wolf, S.J. Johnston, J.E. Raymond, D.E.J. Linden, Neural correlates of enhanced visual short-term memory for angry faces: An fMRI study, PLoS One. 3 (2008). https://doi.org/10.1371/journal.pone.0003536.
[18] M.C. Jackson, D.E.J. Linden, J.E. Raymond, "Distracters" do not always distract: Visual working memory for angry faces is enhanced by incidental emotional words, Front. Psychol. 3 (2012) 1–9. https://doi.org/10.3389/fpsyg.2012.00437.
[19] M.C. Jackson, D.E.J. Linden, J.E. Raymond, Angry expressions strengthen the encoding and maintenance of face identity representations in visual working memory, Cogn. Emot. 28 (2014) 278–297. https://doi.org/10.1080/02699931.2013.816655.
[20] H.J. Lee, Y.S. Cho, Memory facilitation for emotional faces: Visual working memory trade-offs resulting from attentional preference for emotional facial expressions, Mem. Cogn. 47 (2019) 1231–1243. https://doi.org/10.3758/s13421-019-00930-8.
[21] S.C. Linden, M.C. Jackson, L. Subramanian, D. Healy, D.E.J. Linden, Sad benefit in face working memory: An emotional bias of melancholic depression, J. Affect. Disord. 135 (2011) 251–257. https://doi.org/10.1016/j.jad.2011.08.002.
[22] T. Maran, P. Sachse, M. Furtner, From specificity to sensitivity: Affective states modulate visual working memory for emotional expressive faces, Front. Psychol. 6 (2015) 1–16. https://doi.org/10.3389/fpsyg.2015.01297.
[23] P. Sessa, R. Luria, A. Gotler, P. Jolicœur, R. Dell'acqua, Interhemispheric ERP asymmetries over inferior parietal cortex reveal differential visual working memory maintenance for fearful versus neutral facial identities, Psychophysiology. 48 (2011) 187–197. https://doi.org/10.1111/j.1469-8986.2010.01046.x.
[24] P. Sessa, A.S. Lomoriello, R. Luria, Neural measures of the causal role of observers' facial mimicry on visual working memory for facial expressions, Soc. Cogn. Affect. Neurosci. 13 (2018) 1281–1291. https://doi.org/10.1093/scan/nsy095.
[25] L. Simione, L. Calabrese, F.S. Marucci, M.O. Belardinelli, A. Raffone, F.A. Maratos, Emotion based attentional priority for storage in visual short-term memory, PLoS One. 9 (2014). https://doi.org/10.1371/journal.pone.0095261.
[26] S. Spotorno, M. Evans, M.C. Jackson, Remembering who was where: A happy expression advantage for face identity-location binding in working memory, J. Exp. Psychol. Learn. Mem. Cogn. 44 (2018) 1365–1383. https://doi.org/10.1037/xlm0000522.
[27] D. Švegar, I. Kardum, M. Polič, Happy face superiority effect in change detection paradigm, Psihol. Teme. 22 (2013) 249–269.
[28] P.M.J. Thomas, M.C. Jackson, J.E. Raymond, A threatening face in the crowd: Effects of emotional singletons on visual working memory, J. Exp. Psychol. Hum. Percept. Perform. 40 (2014) 253–263. https://doi.org/10.1037/a0033970.
[29] W. Xie, H. Li, X. Ying, S. Zhu, R. Fu, Y. Zou, Y. Cui, Affective bias in visual working memory is associated with capacity, Cogn. Emot. 31 (2017) 1345–1360. https://doi.org/10.1080/02699931.2016.1223020.
[30] L. Zhou, M. Liu, B. Ye, X. Wang, Q. Liu, Sad expressions during encoding enhance facial identity recognition in visual working memory in depression: Behavioural and electrophysiological evidence, J. Affect. Disord. 279 (2021)

630–639. https://doi.org/10.1016/j.jad.2020.10.050.

[31] P. Ekman and W. V. Friesen, Measuring facial movement, Environmental Psychology and Nonverbal Behavior, vol. 1, no. 1, pp. 56–75, 1976.

[32] D. Lundqvist, A. Flykt, and A. Öhman, The Karolinska directed emotional faces (KDEF), CD ROM from Department of Clinical Neuroscience, Psychology section, Karolinska Institutet,1998.

[33] D. Lundqvist, J.E. Litton, The Averaged Karolinska Directed Emotional Faces – AKDEF, CD ROM from Department of Clinical Neuroscience, Psychology section, Karolinska Institute, 1998.

[34] N. Tottenham, J.W. Tanaka, A.C. Leon, T. McCarry, M. Nurse, T. a. Hare, D.J. Marcus, A. Westerlund, B.J. Casey, C. Nelson, The NimStim set of facial expressions: Judgements from untrained research participants, Psychiatry Res. 168 (2009) 242–249. https://doi.org/10.1016/j.psychres.2008.05.006.

[35] F. Solina, P. Peer, B. Batagelj, S. Juvan, J. Kovac Color-based face detection in the 15 seconds of fame art installation Proceedings of Mirage INRIA Rocquencourt France (2003), p. 38 -47 http://web.mit.edu/emeyers/www/face_databases.html

[36] O. Langner, R. Dotsch, G. Bijlstra, D.H.J. Wigboldus, S.T. Hawk, A.D. Van Knippenberg, Presentation and validation of the Radboud Faces Database, Cogn. Emot. 24 (2010) 1377–1388.

[37] M.W. Kim, J.S. Choi, Y.S. Cho, The Korea University Facial Expression Collection (KUFEC) and semantic differential ratings of emotion, Korean J Psychol Gen. 30 (2011) 1189–2111.

[38] M. Liu, W. Hou, C. Yu, The Initial Establishment of Chinese Facial Expression of Emotion System used in Research of Emotional Disorder, Psychol. Explor. (2012) 8.
